# Arginine 16 Glycine Polymorphism in **β**2-Adrenergic Receptor Gene is Associated with Obesity, Hyperlipidemia, Hyperleptinemia, and Insulin Resistance in Saudis

**DOI:** 10.1155/2012/945608

**Published:** 2012-09-27

**Authors:** Maha H. Daghestani, Arjumand Warsy, Mazin H. Daghestani, Ali N. Al-odaib, Abdelmoneim Eldali, Nadia A. Al-Eisa, Sawsan A. Omer, Zeinab K. Hassan

**Affiliations:** ^1^Department of Zoology, Female Center for Scientific and Medical Colleges, King Saud University, P.O. Box 22452, Riyadh 11495, Saudi Arabia; ^2^Department of Genetics, King Faisal Specialist Hospital and Research Center, P.O. Box 3354, Riyadh 11211, Saudi Arabia; ^3^Department of Biochemistry, Female Center for Scientific and Medical Colleges, King Saud University, P.O. Box 22452, Riyadh 11495, Saudi Arabia; ^4^Department of Obstetrics and Gynecology, Umm Al-Qura University, P.O. Box 424, Makkah 21955, Saudi Arabia; ^5^Department of Biostatistics, Epidemiology & Scientific Computing, King Faisal Specialist Hospital and Research Center MBC 03, P.O. Box 3354, Riyadh 11211, Saudi Arabia

## Abstract

*Background*. Several studies have shown an association between codon 16 polymorphism of the **β**2AR gene and obesity. *Methods*. We studied the association between Arg16Gly polymorphism and obesity and its influence on anthropometric parameters, lipids, insulin resistance and leptin in Saudi individuals. The study group included 329 individuals (males: 109 and females: 220). Metabolic parameters, including glucose, lipids, insulin, and leptin were analyzed and anthropometric parameters including waist and hip circumference, waist/hip (W/H) ratio, and body mass index (BMI) were measured and HOMA-IR was calculated. Genotyping was conducted by DNA sequencing of 353 bp fragments, carrying the Arg16Gly polymorphic site. *Results and Conclusion*. Overweight and obese subjects had a significantly higher frequency of Gly16 (0.375 and 0.38, resp.) compared with normal-weight subjects (0.200). In addition, subjects carrying Gly16 allele regardless of their BMI had greater waist and hip circumference, W/H ratio, plasma lipids, leptin, glucose level, and insulin resistance as judged from the HOMA-IR, compared to those with the wild-type allele. The findings of this study show a significant association between the Arg16Gly polymorphism in **β**2AR gene and the development of insulin resistance, overweight, and obesity in Saudi populations with an influence on the levels of lipid and leptin.

## 1. Introduction

Obesity is a global pandemic with multifactorial etiology, where genetic, behavioral, and environmental factors contribute to its development [1]. The search for obesity genes has been actively pursued for the last two decades. Owing to the polygenic nature of obesity, several genetic loci have been implicated in susceptibility to obesity development, under the influence of environmental and behavioral factors [2, 3]. For over two decades, interest has been directed towards the genes involved in the regulation of catecholamine function, due to the central role played by catecholamine in the regulation of energy expenditure [4]. One such group of genes is the adrenergic receptors genes (*α* and *β*), as catecholamines bind to *α*- and *β*-adrenergic receptors; they regulate body fat accumulation and account for energy expenditure. This regulation is in part affected by the stimulation of lipid mobilization through lipolysis activation in fat cells [5, 6]. The *β*2-adrenergic receptors (*β*2ARs) are G-protein coupled receptors, widely distributed in the body. Activation of *β*2ARs expressed in adipocytes mediates lipolysis, while stimulation of *α*2-adrenergic receptors (*α*2ARs) inhibits lipolysis [7]. In addition to lipolysis, they also mediate vaso and bronchial dilation. Insulin is also an important inhibitor of catecholamine-stimulated lipolysis as it reduces the *β*2AR effects of epinephrine and activates *α*2AR in adipocytes [4]. 

Several polymorphisms of the human *β*2AR gene have been described [8, 9]. The *β*2AR gene is highly polymorphic and single nucleotide polymorphisms (SNPs) are known to affect *β*2-receptor affinity, signal transduction, and cellular trafficking *in vitro* [10, 11]. Studies on some ethnic groups have shown that a common polymorphism occurring in codon 16 of the *β*2AR gene is significantly associated with obesity, insulin resistance, and dyslipidemia [12–24], though reports from other populations contradict these results [24–27]. Some of these studies report a gender-specific association.

In the Saudi population, obesity occurs at a high prevalence [29–31] and several genetic loci linked to the development of obesity have been investigated [32, 33]. In an attempt to investigate the association of the common Arg16Gly *β*2AR polymorphism with obesity in Saudi subjects, we conducted a case-control study on normal-weight, overweight and obese individuals. We included leptin a 16 kDa nonglycosylated polypeptide, referred to as the obesity hormone, which plays an important role in the regulation of body fat [34, 35]. The secretion of leptin is affected by food intake, total body fat, and serum levels of several hormones [36], including insulin, and to a lesser extent other peptide hormones. We attempted to investigate the influence of Arg16Gly *β*2AR polymorphism on the leptin levels. This paper describes the results of our studies and shows correlation of the polymorphism with anthropometric and metabolic parameters obtained in the normal, overweight, and obese Saudis.

## 2. Materials and Methods

### 2.1. Study Group

This study included 329 unrelated Saudi men (*n* = 109; 33.1%) and women (*n* = 220; 66.9%), with ages ranging from 18 to 36 years. To avoid selection bias, the individuals were randomly enrolled from a nationwide population attending King Saud University as students or staff members and were individuals who volunteered to be included in the study. Informed written consent was obtained from all study subjects before participation. The study was approved by the local ethics committee of the university (no. 8/27/127228).

### 2.2. Anthropometric and Biochemical Measurements

Anthropometric measurements included BMI (kg/m^2^), waist and hip circumference, and waist-to-hip ratio (WHR). For laboratory studies, blood was sampled in the morning between 08.00 h and 09.00 h after an overnight fast. 5 mL of blood was drawn in plain red-top tubes for the determination of leptin, insulin, and lipids and 2 mL was drawn in fluoride tubes (gray top) for glucose estimation. 5 mL of blood was collected in EDTA-coated tubes for DNA extraction. All blood samples for each subject were immediately centrifuged, and plasma, serum, and buffy coat were stored at −80°C until analysis. Plasma glucose was determined in duplicate by a glucose-oxidase method adapted to an autoanalyzer (Hitachi 704, Boehringer Mannheim, Germany). Total cholesterol, triglycerides, high-density lipoprotein (HDL-cholesterol) and low-density lipoprotein (LDL-cholesterol) were determined by enzymatic methods using commercial kits (Boehringer Mannheim). Plasma insulin and leptin concentrations were determined by radioimmunoassay (RIA; Human Insulin-Specific RIA kit and Human Leptin RIA kit, respectively, Linco Research, St Louis, MO). The value of HOMA-IR was calculated using the standardized formula [37]: glucose (mmol/L) × insulin (mU/L)/22.5.

### 2.3. Genotyping of *β*2AR Polymorphism

The genomic DNAs of 329 subjects were extracted from peripheral blood leukocytes using Gentra Systems Kit (Minneapolis, MN, cat no. D5500). The DNA fragment containing codon 16 of the *β*2AR gene was amplified by polymerase chain reaction (PCR) using a sense primer (5′-AAGCTGAGTGTGCAGGACGA-3′) and an antisense primer (5′-AGACGCTCGAACTTGGCAAT-3′) designed using PRIMER3 program. The PCR conditions, standardized in our laboratory, consisted of an initial denaturation step at 95°C for 15 minutes, followed by 34 cycles of denaturation at 95°C for 1 minute, annealing at 64°C for 1 minute, and extension at 72°C for 1 minute, with a final extension of 10 minutes at 72°C. With these primers, a PCR product of 353 bp was obtained on agarose gel electrophoresis. Nucleotide sequencing was carried out by the ABI Big Dye Terminator protocol using ABI 3100-Avant Genetic Analyzer.

### 2.4. Statistical Analysis

All analyses were run using the Stat-View Program for Windows (version 8.0; SAS) and SPSS (Version 18). Data for males and females were separately analyzed and values for the total population were also obtained. The data are presented as mean ± SEM. The comparisons between overweight, obese subjects, and matched normal-weight controls were carried out using the independent *t*-test and ANOVA test. The distributions of the age, BMI, anthropometric measurements, lipid profile, fasting serum insulin, HOMA-IR, leptin, and glucose in the study subjects according to their *β*2AR gene were compared by the *U* Mann-Whitney test. Multivariable logistic regression was used to study the effect of the *β*2AR genotype on BMI. Correlation analyses were carried out and Pearson's correlation coefficient (*r*) and the *P* value were obtained. Frequency distribution analysis was performed. Genotype and allele frequencies were calculated. Significance of the difference in the results of different groups was obtained using the chi-square test. Frequencies of the different genotypes and alleles in different groups and between males and females were compared. Relative risk was estimated by the odds ratios (ORs) and their 95% confidence intervals (CIs). A probability value *P* ≤ 0.05 was considered statistically significant. 

## 3. Results

The results of BMI were used to classify the male and female population as normal-weight (BMI: ≤24.9 kg/m^2^), overweight (BMI: 25–29.9 kg/m^2^), and obese (BMI: ≥30 kg/m^2^) individuals. There were 35, 28, and 46 males and 80, 40, and 100 females in the normal-weight, overweight, and obese groups, respectively. The anthropometric data of all subjects including males and females are presented in Table 1. There were slight, nonsignificant differences in the results of the males and females. Comparison of the anthropometric characteristics between normal-weight, overweight, and obese subjects showed that all parameters were significantly higher in overweight and obese subjects compared with the normal-weight individuals, both in the males and females.

Table 2 presents the results of glucose, plasma lipids, insulin, HOMA-IR, and leptin, in the males and females, in the three different weight groups. The male and female results showed some minor non-significant differences; however, levels of leptin were significantly higher in the females compared to the males. In both males and females, the results of these parameters varied significantly between the normal-weight, overweight and obese groups, with significance reaching values <0.0001. 

The frequency of Arg16Gly alleles and genotypes was calculated. In both the male and female groups, overweight and obese subjects had a significantly higher genotype and allele frequency of Gly16 compared with normal-weight subjects. The genotype and allele frequencies in the males, females, and the total group are presented in Table 3 with the OR, CI, *χ* ^2^, and *P* value. The study group was separated according to their Arg16Gly genotypes and the phenotypic characteristics were obtained. The results of the different parameters in the different genotypes are presented in Table 4. This table shows that the subjects carrying Gly16 in homozygous state had a greater BMI, waist and hip circumference, W/H ratio, cholesterol, triglyceride, LDL-C, HOMA-IR, and plasma leptin compared with those with the Arg16 allele, and the difference was significant (*P* < 0.05). Correlation studies were carried out between the different parameters separately in the different genotypes. Significant correlations were encountered, where BMI, waist, hip, and waist/hip ratio correlated positively and significantly with cholesterol, triglyceride, and leptin in the individuals with all genotypes (*P* < 0.0001) (results not shown). However, with glucose the positive correlation was only in the wild-type Arg/Arg individuals (*P* < 0.0001), while there was no correlation in the Arg/Gly and Gly/Gly genotypes (*P* > 0.05) between glucose and the other parameters (Table 5), except for HOMA-IR which showed a positive and significant correlation with glucose in each genotype. 

## 4. Discussion 

Obesity is a major problem in the Saudi population and, to an extent, is believed to be due to the rapid transition in the lifestyles of the affluent. A sedentary lifestyle linked to the warm climate, in addition to an increased consumption of carbohydrates, along with a genetic predisposition, further contributes to the development of obesity [27, 29–31]. Some studies have been conducted on the leptin receptor and ghrelin gene and the polymorphisms have been associated with development of obesity in Saudis [32, 33]. However, as in other populations and in line with other multifactorial disorders, studies on identification of genes contributing to the development of obesity still require additional extensive investigations. The picture is further complicated as populations show differences in the association between different gene loci, different polymorphisms, and SNPs and development of obesity. This entails the search for susceptibility gene loci for each population and in each ethnic group. 

Our earlier studies on Gln/Glu polymorphism at position 27 in *β*2AR showed an effect of this genetic variation on lipid phenotypes, insulin, and leptin levels in overweight/obese individuals where BMI, triglycerides, leptin, and insulin levels were higher in the Saudi individuals carrying the Glu/Glu genotype. However, there were no differences in the genotype or allele frequency of the Gln and Glu variants in normal-weight, overweight, and obese individuals [28]. As a continuation of the earlier study we investigated the Arg/Gly polymorphism at position 16 in the *β*2AR in the same Saudi cases and controls and our results show interesting associations.

Applying Hardy-Weinberg equation, our results showed that in the normal control group, the Arg16Gly *β*2AR genotypes were in Hardy-Weinburg equilibrium, while overweight and obese Saudi subjects have a higher frequency of the Gly16 allele, and the subjects who carry Gly16 homozygote genotype have greater BMI and waist-to-hip ratios (abdominal obesity) associated with higher levels of cholesterol, triglyceride, LDL-C, serum leptin, and HOMA-IR. The presence of *β*2AR Gly/Gly genotype and Gly16 allele increases the relative risk of developing overweight and obesity by almost 2.5 times as seen from the OR and the difference in the number of overweight and obese was significantly higher in those with this allele. Insulin resistance is significantly higher in individuals carrying the Gly/Gly genotype compared to the Arg/Arg genotype and this could be one of the etiological factors leading to higher prevalence of obesity in this genotype. The Arg allele was protective against the development of these conditions with an OR of around 0.4. In this study we have observed that the Gly16 alleles of the *β*2AR genes are related to abdominal obesity and hypertriglyceridaemia, hyperinsulinaemia, hyperleptinaemia, and insulin resistance in Saudis. We suggest that this may be the result of decreased binding of the catecholamines to the *β*2AR with the Gly16 allele. It must be pointed out that this study was conducted on a small sample size and larger studies are required to confirm the statistical significance of this association.

Catecholamines play an essential role in energy expenditure and lipolysis. Binding to *β*2AR mediates lipolysis and this eventually results in weight loss. It is possible that substitution of Arg16, a basic amino acid, by Gly a neutral amino acid, influences the binding of catecholamines to the *β*2AR and hence prevents lipolysis. Catecholamine-stimulated whole-body lipolysis and lipolysis in subcutaneous adipocytes are blunted in obesity [38], thereby limiting lipid mobilization and favoring fat accumulation. This is confirmed by both *in vitro* and *in vivo* data, which suggests that catecholamine resistance, restricted to the *β*2-adrenoceptor, may be due to a decreased number and/or function of *β*2-adrenoceptors [39]. It implies that alteration in *β*2AR structure due to a single amino acid change may contribute to catecholamine resistance and hence overweight and obesity. Masuo et al. [18] have reported that the Gly16 mutation of the *β*2AR gene is associated with increased insulin resistance, adiposity, and blood pressure accompanied by higher plasma norepinephrine and leptin levels. Ukkola et al. [15] found that gene-to-gene interactions among the *α*_2_-, *β*_2_-, and *β*_3_-adrenergic receptor genes contributed to the phenotypic variability in the abdominal obesity and plasma lipid and lipoprotein in a family study in Québec. However, there is considerable debate on this association, because there have been several studies showing no significant association between *β*2AR gene polymorphism and obesity [23–25, 33]. Hayakawa et al. [26] reported that Arg16Gly polymorphisms of the *β*2AR gene are not a major contributing factor to obesity, blood pressure, serum lipid levels, uric acid, or free fatty acid levels in 210 Japanese men. Similarly, Kim et al. [23] found that the Arg16Gly polymorphisms of the *β*2AR gene are not major contributing factors to obesity in Korean subjects.

In the present study, several significant correlations were observed between the anthropometric measures and biochemical and hormonal parameters. However, these correlations seem to be related to the Arg16Gly genotype (Table 4). All investigated parameters had a higher value in the homozygotes for Gly allele (Gly/Gly) when compared with the wild-type allele (Arg/Arg). Cholesterol, triglyceride, and leptin levels increased positively with high BMI, waist, hip, and waist/hip ratio in the wild-type Arg/Arg, Arg/Gly and Gly/Gly genotypes. However, an interesting correlation between glucose and the other parameters was observed when the different genotypes were compared. Whereas glucose showed a significant positive correlation with age, BMI, waist, hip, W/H ratio, cholesterol, triglyceride, LDL, leptin, and insulin, (*P* < 0.001) and a negative correlation with HDL-cholesterol (*P* < 0.001) in the wild-type genotype (Arg/Arg), the majority of these correlations were lost in the Gly/Gly and Arg/Gly genotype, in particular those with the anthropometric measures (*P* > 0.05). This would suggest that while glucose levels can be reduced by reducing BMI, weight, waist, and W/H ratio in wild-type Arg/Arg individuals; this is not the case in those with Arg/Gly and Gly/Gly individuals. Hence, these individuals are at risk of development of hyperglycemia and diabetes mellitus, regardless of whether they are normal weight, overweight, or obese. Even the biochemical parameters studied during this investigation showed no correlation to glucose in the Gly/Gly individuals and they had elevated levels of each of these parameters compared to the individuals with Arg/Arg genotype. Thus, a correlation between different biochemical and the anthropometric parameters seem to be related to the polymorphisms of the *β*2AR gene. Such an observation has not been previously reported. Another interesting finding was that when the Arg/Arg, Arg/Gly and Gly/Gly genotypes were analyzed for the number of people suffering from insulin resistance, in each genotype, using a cut-off value of 2.5 of HOMA-IR, none of the individuals in the Arg/Arg genotype had insulin resistance (0/189); 30/68 (55.9%) people with Arg/Gly genotype had insulin resistance and 39/70 (55.8%) of the Gly/Gly genotype were suffering from insulin resistance. This finding indicates two things. Firstly, the excess of insulin resistance could be the cause of higher prevalence of overweight and obesity in the Arg/Gly and Gly/Gly genotypes. Secondly, this also indicates a dominant effect of the Gly allele; that is, once present, whether in homozygotes or heterozygotes, it has an effect on the phenotype. Further studies are needed to confirm this hypothesis. 

This study has shown an interesting association between the Arg16Gly polymorphism and weight gain, waist/hip ratio, and lipid profiles. However, the main limitation of the study is the small sample size studied and the association needs to be confirmed by conducting a study on a larger sample size. Furthermore, haplotype construction using the Arg16Gly and the Glu27Gln polymorphic sites may reveal additional association or otherwise with obesity phenotype.

In summary, a complex picture is emerging as more and more population-based studies are being carried out on the genes contributing to development of obesity. Among the Arabs, the picture may be different when compared to Caucasians and the population of the Far East. Hence, further studies in larger populations such as Arabs from different regions are required to verify these results.

## 5. Conclusions

In conclusion, our results show that Arg16Gly polymorphism plays a major role in pathogenesis of obesity, affecting lipid phenotypes, leptin levels, and insulin resistance, in individuals with different genotypes. 

## Figures and Tables

**Table 1:** Anthropometric characteristics of normal-weight, overweight, and obese male and female subjects (from [28]).

Variables	Normal weight (*n* = 115) mean ± SE	Overweight (*n* = 68) mean ± SE	Obese (*n* = 146) mean ± SE	*P* value
Age (yr)				
M	25.9 ± 0.7	27.3 ± 0.67	26.7 ± 0.73	0. 056
F	24.50 ± 0.50	24.4 ± 0.70	26.7 ± 0.54	
T	24.90 ± 0.44	25.56 ± 0.54	26.70 ± 0.43	
BMI (kg/m^2^)				
M	21.27 ± 0.34	26.4 ± 0.27	32.90 ± 0.80	0.0001
F	21.4 ± 0.20	27.3 ± 0.20	35.80 ± 0.60	
T	21.33 ± 0.19	26.93 ± 0.17	34.89 ± 0.50	
Waist (cm)				
M	74.20 ± 1.53	86.10 ± 1.40	110.50 ± 2.20	0.0001
F	67.30 ± 0.90	84.40 ± 1.23	100.60 ± 1.38	
T	70.82 ± 0.79	85.13 ± 0.93	103.72 ± 1.23	
Hip (cm)				
M	97.60 ± 1.20	100.80 ± 1.17	122.20 ± 2.00	0.0001
F	95.50 ± 0.80	102.20 ± 1.10	118.50 ± 1.40	
T	96.13 ± 0.68	102.22 ± 0.82	119.68 ± 1.15	
W/H Ratio				
M	0.76 ± 0.02	0.86 ± 0.01	0.90 ± 0.01	0.0001
F	0.72 ± 0.01	0.82 ± 0.02	0.85 ± 0.007	
T	0.73 ± 0.006	0.84 ± 0.006	0.87 ± 0.006	

Note: Values are mean ± SEM. Abbreviations—BMI: body mass index; W/H: waist/ hip ratio; M: male; F: female; T: total.

**Table 2:** Comparisons of clinical parameters amongst normal-weight, overweight, and obese males and females (from [28]).

Variables	Sex*	Normal weight (*n* = 115) mean ± SE	Overweight (*n* = 68) mean ± SE	Obese (*n* = 146) mean ± SE	*P* value
Cholesterol (mmol/L)	M	3.31 ± 0.07	4.4 ± 0.15	4.1 ± 0.11	0.0001
	F	3.5 ± 0.05	4.05 ± 0.12	4.5 ± 0.09	
	T	3.43 ± 0.04	4.19 ± 0.09	4.4 ± 0.07	

Triglyceride (mmol/L)	M	0.79 ± 0.05	1.15 ± 0.08	1.43 ± 0.07	0.0001
	F	0.72 ± 0.05	1.03 ± 0.06	1.24 ± 0.04	
	T	0.74 ± 0.02	1.082 ± 0.05	1.302 ± 0.04	

HDL cholesterol (mmol/L)	M	1.28 ± 0.06	1.21 ± 0.07	1.08 ± 0.04	
	F	1.34 ± 0.04	1.17 ± 0.05	1.06 ± 0.03	0.0001
	T	1.32 ± 0.03	1.19 ± 0.04	1.07 ± 0.024	

LDL cholesterol (mmol/L)	M	1.58 ± 0.09	2.25 ± 0.15	2.4 ± 0.11	0.0001
	F	1.53 ± 0.06	1.94 ± 0.11	2.6 ± 0.07	
	T	1.55 ± 0.05	2.07 ± 0.89	2.56 ± 0.06	

Leptin (ng/mL)	M	3.10 ± 0.17**	9.91 ± 0.38**	17.6 ± 0.86**	0.0001
	F	12.20 ± 0.40**	22.84 ± 0.97**	44.60 ± 1.85**	
	T	9.42 ± 0.48	17.51 ± 0.97	36.13 ± 1.67	

Fasting insulin (pmol/L)	M	62.60 ± 4.23	67.07 ± 5.27	111.90 ± 5.6	0.0001
	F	54.85 ± 2.33	75.84 ± 6.26	103.30 ± 3.64	
	T	57.21 ± 2.09	72.23 ± 4.28	106.03 ± 3.07	

HOMA-IR	M	1.96 ± 0.14	2.4 ± 0.21	3.8 ± 0.18	0.0001
	F	1.66 ± 0.08	2.2 ± 0.17	3.37 ± 0.13	
	T	1.75 ± 0.07	2.35 ± 0.14	3.45 ± 0.11	

Fasting glucose (mmol/L)	M	4.84 ± 0.07	5.16 ± 0.05	5.06 ± 0.09	0.0001
	F	4.65 ± 0.05	4.97 ± 0.08	5.06 ± 0.06	
	T	4.71 ± 0.041	5.051 ± 0.057	5.06 ± 0.05	

Note: Values are mean ± SEM. Abbreviations—BMI: body mass index; W/H: waist hip ratio; M: male; F: female; T: total; *Statistically significant difference between the male and female. *Total number: control: male = 35; female = 80; total = 115. *Total overweight: male = 28; female = 40; total = 68. *Total obese: male = 46; female = 100; total = 146. SE: standard error of the mean, **: the difference in the results of males and females is statistically significant.

**Table 3:** Distribution of the genotypes, allele frequencies, and odd ratio of the *β*2 Arg16Gly polymorphism in total control, overweight, and obese subjects, separately in males and females.

Group	No.	Genotype frequency*				Allele frequency**		
		Arg*∖*Arg	Arg*∖*Gly	Gly*∖*Gly	*χ* ^2^/*P*	Arg	Gly	*χ* ^2^/*P*
Males								
Obese	46	25 (54.3)	9 (19.56)	12 (26.1)	3.82/0.43	59	33	5.02/0.08
Overweight	28	14 (50)	7 (25)	7 (25)		35	21	
Control	35	25 (71.4)	5 (14.28)	5 (14.28)		55	15	
Females								
Obese	100	51 (51)	20 (20)	29 (29)	12.7/0.013	122	78	17.4/<0.0001
Overweight	40	20 (50)	10 (25)	10 (25)		50	30	
Control	80	56 (70)	17 (21.25)	7 (8.78)		129	31	
Total group								
Obese	146	76 (50.1)	29 (19.9)	41 (28.1)	15.5/0.002	181	111	22.1/<0.0001
Overweight	68	34 (50)	17 (25)	17 (25)		85	51	
Control	115	81 (70.4)	22 (19.1)	12 (10.4)		184	46	

*For genotype frequency:

odds ratio (95% CI) control versus OW: 3.38 (1.456, 7.822);

odds ratio (95% CI) control versus Obese: 2.441 (0.667, 2.971);

*P* value for odds ratio in control versus overweight and control versus obese: *P* = 0.0001;

*statistically significant difference between the three groups (obese, overweight and normal);

**For allele frequency:

odds ratio (95% CI) control versus OW: 0.60 (0.46, 0.78) (*P* = 0.02);

odds ratio (95% CI) control versus obese: 0.70 (0.60, 0.810) (*P* = 0.001);

*allele frequency differs significantly in the different groups (*P* < 0.0001).

M: male; F: female; T: total; OW: overweight; CI: confidence interval.

**Table 4:** Association of *β*2AR genotype in codon 16 with phenotypic characteristics of the subjects.

Variables	Arg*∖*Arg (*n* = 191) mean ± SE	Arg*∖*Gly (*n* = 68) mean ± SE	Gly*∖*Gly (*n* = 70) mean ± SE	*P* value
BMI (kg/m^2^)	27.15 ± 0.51	28.82 ± 0.85	31.88 ± 0.91	0.0001
Waist (cm)	85.32 ± 1.32	89.24 ± 2.14	95.89 ± 2.28	0.0001
Hip (cm)	105.48 ± 1.05	108.32 ± 1.71	113.81 ± 1.97	0.0001
W/H ratio	0.80 ± 0.006	0.82 ± 0.01	0.84 ± 0.01	0.025
Cholesterol (mmol/L)	3.90 ± 0.06	4.03 ± .09	4.32 ± .11	0.002
Triglyceride (mmol/L)	0.99 ± 0.03	1.09 ± 0.05	1.21 ± 0.06	0.003
HDL-cholesterol (mmol/L)	1.20 ± .02	1.19 ± .05	1.11 ± .04	0.141
LDL-cholesterol (mmol/L)	2.013 ± 06	2.11 ± .09	2.34 ± .088	0.012
Leptin (ng/mL)	19.80 ± 1.11	24.96 ± 2.59	29.56 ± 2.64	0.0001
Fasting insulin (pmol/L)	76.57 ± 2.74	86.62 ± 4.61	92.21 ± 4.93	0.09
Fasting glucose (mmol/L)	4.91 ± .04	4.92 ± 06	5.03 ± 05	0.24
HOMA-IR	2.40 ± 0.09	2.75 ± 0.15	3.00 ± 0.17	0.003

Abbreviations—BMI: body mass index; W/H: waist hip ratio.

**Table 5:** Correlation between fasting glucose and the anthropometric parameters and lipids in individuals with different Arg16Gly genotypes.

Correlation between glucose and different parameters in different genotypes		BMI kg/m^2^	Waist cm	Hip cm	W/H ratio	Chol mmol/L	TAG mmol/L	HDL mmol/L	LDL mmol/L	Leptin ng/mL	Insulin pmol/L	Homa IR
Arg/Arg	*r*	0.28**	0.39**	0.26**	0.42**	0.24**	0.31**	−0.27**	0.30**	0.19**	0.26**	0.47**
(189)	*P*	0.000	0.000	0.000	0.000	0.001	0.0001	0.0001	0.0001	0.008	0.0001	0.000

Arg/Gly	*r*	0.15	0.21	0.14	0.25*	0.42**	−0.03	0.13	0.47**	0.10	0.17	0.38**
(68)	*P*	0.24	0.08	0.25	0.04	0.000	0.79	0.29	0.000	0.41	0.17	0.001

Gly/Gly	*r*	0.18	0.16	0.14	0.12	0.003	0.11	−0.23	0.01	0.03	0.31**	0.44**
( 70)	*P*	0.14	0.18	0.26	0.31	0.98	0.36	0.06	0.92	0.83	0.009	0.000

Abbreviations: BMI: body mass index; W/H: waist hip ratio; *r*: pearson correlation coefficient; Chol: cholesterol; TAG: triglyceride; HDL: HDL-cholesterol; LDL: LDL-cholesterol; **significant at the 0.0001 level.

## References

[1] Bouchard C (1991). Current understanding of the etiology of obesity: genetic and nongenetic factors. *American Journal of Clinical Nutrition*.

[2] Spiegelman BM, Flier JS (1996). Adipogenesis and obesity: rounding out the big picture. *Cell*.

[3] Rankinen T, Zuberi A, Chagnon YC (2006). The human obesity gene map: the 2005 update. *Obesity*.

[4] Lafontan M, Berlan M (1993). Fat cell adrenergic receptors and the control of white and brown fat cell function. *Journal of Lipid Research*.

[5] Lonnqvist F, Wahrenberg H, Hellstrom L, Reynisdottir S, Arner P (1992). Lipolytic catecholamine resistance due to decreased *β*2-adrenoceptor expression in fat cells. *Journal of Clinical Investigation*.

[6] Enocksson S, Shimizu M, Lonnqvist F, Nordenstrom J, Arner P (1995). Demonstration of an *in vivo* functional *β*
_3_-adrenoceptor in man. *Journal of Clinical Investigation*.

[7] Barbe P, Millet L, Galitzky J, Lafontan M, Berlan M (1996). In situ assessment of the role of the *β*1-, *β*2- and *β*3-adrenoceptors in the control of lipolysis and nutritive blood flow in human subcutaneous adipose tissue. *British Journal of Pharmacology*.

[8] Green SA, Turki J, Hall IP, Liggett SB (1995). Implications of genetic variability of human *β*2-adrenergic receptor structure. *Pulmonary Pharmacology*.

[9] Liggett SB (2000). Pharmacogenetics of beta-1- and beta-2-adrenergic receptors. *Pharmacology*.

[10] Green SA, Turki J, Innis M, Liggett SB (1994). Amino-terminal polymorphisms of the human beta 2-adrenergic receptor impart distinct agonist-promoted regulatory properties. *Biochemistry*.

[11] Green SA, Cole G, Jacinto M, Innis M, Liggett SB (1993). A polymorphism of the human *β*2-adrenergic receptor within the fourth transmembrane domain alters ligand binding and functional properties of the receptor. *Journal of Biological Chemistry*.

[12] Ishiyama-Shigemoto S, Yamada K, Yuan X, Ichikawa F, Nonaka K (1999). Association of polymorphisms in the *β*2-adrenergic receptor gene with obesity, hypertriglyceridaemia, and diabetes mellitus. *Diabetologia*.

[13] Iwamoto N, Ogawa Y, Kajihara S (2001). Gln27Glu *β*2-adrenergic receptor variant is associated with hypertriglyceridemia and the development of fatty liver. *Clinica Chimica Acta*.

[14] Arner P (2001). Genetic variance and lipolysis regulation: implications for obesity. *Annals of Medicine*.

[15] Ukkola O, Rankinen T, Weisnagel SJ (2000). Interactions among the *α*2-, *β*2-, and *β*3-adrenergic receptor genes and obesity-related phenotypes in the Quebec Family Study. *Metabolism*.

[16] Mori Y, Kim-Motoyama H, Ito Y (1999). The Gln27Glu *β*2-adrenergic receptor variant is associated with obesity due to subcutaneous fat accumulation in Japanese men. *Biochemical and Biophysical Research Communications*.

[17] Masuo K, Katsuya T, Fu Y, Rakugi H, Ogihara T, Tuck ML (2005). *β*2-adrenoceptor polymorphisms relate to insulin resistance and sympathetic overactivity as early markers of metabolic disease in nonobese, normotensive individuals. *American Journal of Hypertension*.

[18] Masuo K, Katsuya T, Kawaguchi H (2006). *β*
_2_-Adrenoceptor polymorphisms relate to obesity through blunted leptin mediated sympathetic activation. *American Journal of Hypertension*.

[19] Large V, Hellström L, Reynisdottir S (1997). Human beta-2 adrenoceptor gene polymorphisms are highly frequent in obesity and associate with altered adipocyte beta-2 adrenoceptor function. *Journal of Clinical Investigation*.

[20] Leineweber K, Büscher R, Bruck H, Brodde OE (2004). *β*-Adrenoceptor polymorphisms. *Naunyn-Schmiedeberg’s Archives of Pharmacology*.

[21] Masuo K, Katsuya T, Fu Y, Rakugi H, Ogihara T, Tuck ML (2005). *β*2- and *β*3-adrenergic receptor polymorphisms are related to the onset of weight gain and blood pressure elevation over 5 years. *Circulation*.

[22] Kawaguchi H, Masuo K, Katsuya T (2006). *β*2- and *β*3-adrenoceptor polymorphisms relate to subsequent weight gain and blood pressure elevation in obese normotensive individuals. *Hypertension Research*.

[23] Kim SH, Kim DJ, Seo IA (2002). Significance of *β*2-adrenergic receptor gene polymorphism in obesity and type 2 diabetes mellitus in Korean subjects. *Metabolism*.

[24] Gjesing AP, Andersen G, Burgdorf KS (2007). Studies of the associations between functional *β*2- adrenergic receptor variants and obesity, hypertension and type 2 diabetes in 7,808 white subjects. *Diabetologia*.

[25] Garenc C, Pérusse L, Chagnon YC (2003). Effects of *β*2-adrenergic receptor gene variants on adiposity: the HERITAGE Family Study. *Obesity Research*.

[26] Hayakawa T, Nagai Y, Kahara T (2000). Gln27Glu and Arg16Gly polymorphisms of the *β*2-adrenergic receptor gene are not associated with obesity in Japanese men. *Metabolism*.

[27] Mattevi VS, Zembrzuski VM, Hutz MH (2006). Impact of variation in ADRB2, ADRB3, and GNB3 genes on body mass index and waist circumference in a Brazilian population. *American Journal of Human Biology*.

[28] Daghestani MH, Warsy AA, Daghestani MH (2010). The Gln27Glu polymorphism in *β*2-adrenergic receptor gene is linked to hypertriglyceridemia, hyperinsulinemia and hyperleptinemia in Saudis. *Lipids in Health and Disease*.

[29] Al-Nozha MM, Al-Mazrou YY, Al-Maatouq MA (2005). Obesity in Saudi Arabia. *Saudi Medical Journal*.

[30] Al-Malki JS, Al-Jaser MH, Warsy AS (2003). Overweight and obesity in Saudi females of childbearing age. *International Journal of Obesity*.

[31] Warsy AS, El-Hazmi MAF (1999). Diabetes mellitus, hypertension and obesity—common multifactorial disorders in Saudis. *Eastern Mediterranean Health Journal*.

[32] Daghestani MH (2006). *Levels of leptin and reproductive hormones and detection of leptin receptor gene in Saudi Females with Polycystic ovary syndrome [Ph.D. thesis]*.

[33] Galletti F, Iacone R, Ragone E (2004). Lack of association between polymorphism in the *β*2- adrenergic receptor gene, hypertension, and obesity in the Olivetti Heart Study. *American Journal of Hypertension*.

[34] Kershaw EE, Flier JS (2004). Adipose tissue as an endocrine organ. *Journal of Clinical Endocrinology and Metabolism*.

[35] Havel PJ (2000). Role of adipose tissue in body-weight regulation: mechanisms regulating leptin production and energy balance. *Proceedings of the Nutrition Society*.

[36] Mantzoros CS, Moschos SJ (1998). Leptin: In search of role(s) in human physiology and pathophysiology. *Clinical Endocrinology*.

[37] Matthews DR, Hosker JP, Rudenski AS (1985). Homeostasis model assessment: insulin resistance and *β*-cell function from fasting plasma glucose and insulin concentrations in man. *Diabetologia*.

[38] Reynisdottir S, Wahrenberg H, Carlstrom K, Rossner S, Arner P (1994). Catecholamine resistance in fat cells of women with upper-body obesity due to decreased expression of beta2-adrenoceptors. *Diabetologia*.

[39] Jocken JWE, Blaak EE (2008). Catecholamine-induced lipolysis in adipose tissue and skeletal muscle in obesity. *Physiology and Behavior*.

